# Assessment of Gait and Balance in Elderly Individuals with Knee Osteoarthritis Using Inertial Measurement Units †

**DOI:** 10.3390/s25206288

**Published:** 2025-10-10

**Authors:** Lin-Yen Cheng, Yen-Chang Chien, Tzu-Tung Lin, Jou-Yu Lin, Hsin-Ti Cheng, Chia-Wei Chang, Szu-Fu Chen, Fu-Cheng Wang

**Affiliations:** 1 Department of Physical Medicine and Rehabilitation, Cheng Hsin General Hospital, Taipei 112, Taiwan; a09830886@gmail.com (L.-Y.C.); cute29554416@gmail.com (Y.-C.C.); b101100094@tmu.edu.tw (T.-T.L.); trancequid@gmail.com (J.-Y.L.); 2 Department of Mechanical Engineering, National Taiwan University, Taipei 106, Taiwan; r11522858@ntu.edu.tw (H.-T.C.); r12522849@ntu.edu.tw (C.-W.C.); 3 Department of Physiology and Biophysics, National Defense Medical Center, Taipei 114, Taiwan

**Keywords:** knee osteoarthritis, elderly, balance, inertial measurement unit, angular velocities

## Abstract

Knee osteoarthritis (OA) is a prevalent condition in older adults that often results in impaired gait and balance, increased risk of falls, and reduced quality of life. Conventional clinical assessments may not adequately capture these deficiencies. This study investigated the gait and balance of elderly individuals with knee OA using wearable inertial measurement units (IMUs). Forty-four participants with Kellgren–Lawrence grade 2–3 knee OA (71.23 ± 5.75 years) and forty-five age-matched controls (70.87 ± 4.30 years) completed dynamic balance (balance board), static balance (single-leg stance), ‘timed up and go’ (TUG), and normal walking tasks. Between 2 and 8 IMUs, depending on the task, were placed on the head, chest, waist, knees, ankles, soles, and balance board to record kinematic data. Balance was quantified using absolute angular velocity and linear acceleration, with group differences analyzed by MANOVA and Bonferroni-adjusted univariate tests. The participants with knee OA exhibited greater gait asymmetry, although the difference was not significant. However, they consistently demonstrated higher absolute angular velocities than controls across most body segments during static and dynamic tasks, indicating reduced postural stability. No group differences were observed in TUG performance. These findings suggest that IMU-based measures, particularly angular velocity, are sensitive to balance impairment detection in knee OA. Incorporating IMU technology into clinical assessments may facilitate early identification of instability and guide targeted interventions to reduce fall risk.

## 1. Introduction

Knee osteoarthritis (OA) is one of the most common degenerative joint diseases in the elderly, and is characterized by cartilage degradation, joint space reduction, osteophyte formation, and resultant joint dysfunction [1]. Beyond the hallmark symptoms of pain, stiffness, and reduced mobility, emerging evidence highlights the profound impact of knee OA on balance control, which significantly elevates the risk of falls in this population [2,3]. The key factors contributing to the risk of falls include impaired proprioception, reduced range of motion, and heightened fear of falling. These physical imitations often lead to compensatory gait adaptation and increased postural insecurity, further compromising balance and stability during ambulation [2,3]. Given these clinical implications, precise identification and quantification of balance deficits in patients with knee OA are critical. Such assessments enable the stratification of fall risk and individualized rehabilitation interventions, including targeted exercise regimens and patient education, with the potential to enhance functional outcomes and reduce healthcare expenditure [1].

Balance is a dynamic process that relies on the integrative function of visual, vestibular, and somatosensory inputs along with neuromuscular control and central nervous system processing [4]. This complex system is disrupted by multiple factors in patients with knee OA. Notably, proprioceptive acuity declines due to joint degeneration, impairing the detection of subtle changes in joint position [5]. Concurrently, quadriceps weakness and activity secondary to pain exacerbate deficits in knee stabilization and dynamic balance control [4].

These deficits often result in compensatory movement patterns, such as excessive trunk motion, to maintain balance [5]. However, such strategies may be ineffective and can even destabilize the center of mass, thus increasing the risk of falls, especially during dual tasking and when visual, vestibular, and somatosensory inputs are altered. This instability is particularly concerning in older adult populations because even minor perturbations in balance control can pose a significant risk for falls and injury [6].

The ‘timed up and go’ (TUG) [7] and Berg balance scale [8] are common, function-based scales for assessing mobility as well as static and dynamic balance across functional tasks. Although these tools are widely available and easy to conduct, they provide only categorical or ordinal data and lack the sensitivity to detect subtle postural adjustments. These tests also show ceiling effects in community-dwelling elders [9,10], and provide minimal insight into intersegmental coordination [11]. By contrast, quantitative assessments provide significantly greater precision. Instrumented methods, such as force plates, optical motion capture and surface electromyography yield high-resolution information [12,13,14], but are limited by cost and laboratory settings. Consequently, although quantitative tools offer valuable insights into the mechanisms underlying balance impairment, their accessibility and feasibility in routine clinical practice remain challenging.

In contrast to traditional laboratory-based methods, wearable sensor systems utilizing inertial measurement units (IMUs) have emerged, demonstrating the potential for objective and portable balance assessment in clinical research [15]. These devices, which integrate triaxial accelerometers, gyroscopes, and magnetometers, provide real-time kinematic data on linear acceleration and angular velocity. The ability to attach IMUs to multiple anatomical sites allows the segmental analysis of postural control, offering a more comprehensive outlook than single-site measurements. The reliability and validity of IMUs have been established across a range of static and dynamic conditions [16], enabling their use in real-world settings for studying various populations, including older adults [17,18,19,20] and those with neurological conditions. Research has demonstrated the utility of IMUs in assessing fall risk [18], evaluating postural adaptation, and differentiating between fallers and non-fallers based on sway parameters [19]. A recent systematic review demonstrated that accelerometer-based sensors yield valid and reliable measurements of gait alterations in individuals with knee OA, including gait speed, stride length, range of knee swing, and foot strike and toe-off angles [21]. In addition, a comprehensive scoping review of wearable IMU applications in lower-limb OA reported that most studies positioned sensors on the back or shank and predominantly assessed spatiotemporal, angular, or linear acceleration metrics [22]. Although these systems offer significant advantages, their applications targeting specific populations such as older adults with knee osteoarthritis require further investigation. Existing studies have primarily focused on gait analysis or used a single sensor [23,24], which can fail to capture the complex, multi-segmental movements required for effective balance control, lacking the sensitivity to detect subtle impairments. Therefore, future research should explore multi-site IMU deployment to fully leverage the potential of these devices for a more nuanced understanding of postural stability in this population.

To fill this gap, the present study employs a multi-site IMU setup on several body segments (head, chest, waist, knees, ankles, soles, and balance board) to comprehensively assess gait asymmetry and postural control in older adults with knee OA. Unlike previous work focusing mainly on spatiotemporal gait parameters, our study incorporates both static (e.g., single-leg stance) and dynamic (e.g., balance board) balance tasks and highlights the utility of novel postural indices. Specifically, we use two complementary balance measures—average absolute angular velocity ($J_{\omega }$) and average absolute linear acceleration ($J_{\alpha }$)—to quantify rotational and translational sway during normal walking and under both static and dynamic conditions in elderly individuals with and without knee OA.

Our study advances prior approaches in three key aspects: (i) combining a dynamic task paradigm with the traditional static stance test to capture a broader range of balance performance; (ii) employing segment-level absolute angular-velocity ($J_{\omega }$) together with absolute linear-acceleration indices ($J_{\alpha }$) to provide sensitive, complementary information on postural control; and (iii) enabling early detection of compensatory strategies at joints beyond the knee (e.g., hip and ankle), revealing subtle control deficits often missed by traditional clinical tools. These methodological advances enhance the clinical utility of multi-site IMUs and may inform the design and progression of more targeted rehabilitation programs to mitigate fall risk in high-risk populations.

## 2. Materials and Methods

### 2.1. Study Design

This cross-sectional research was granted with approval by the Institutional Review Board of Cheng Hsin General Hospital in Taiwan (Approval number: (1135)113A-65). A cross-sectional design was chosen as it enables the simultaneous comparison of gait and balance performance in older adults with Knee OA and age-matched healthy controls. This approach provides an effective means of identifying group differences in postural stability and gait asymmetry, although it does not allow cause-and-effect relationships to be drawn. The baseline data obtained in this study will provide a foundation for future intervention trials aimed at evaluating whether targeted rehabilitation programs can modify the detected impairments and compensatory strategies at non-knee joints. Balance-measurement data was collected from all participants after written consent; they all agreed on the use of all information included in the article.

### 2.2. Participants

A total of 89 participants were recruited for this study, consisting of 44 patients clinically diagnosed with knee OA and 45 age-matched healthy controls. We included participants with knee OA if they met the American College of Rheumatology criteria [25], which required both clinical symptoms and objective clinical evidence of osteoarthritis (e.g., radiographic findings). Eligible patients had at least one knee with Kellgren Lawrence grade 2–3 [26]; grade 4 patients were excluded due to severe pain and functional limitations that could compromise the reliability of balance and gait assessments. The control group consisted of healthy older adults without a history of musculoskeletal or neurological disorders that would affect balance or gait.

All participants met the following inclusion criteria: (1) ≥60 years of age, (2) able to walk independently at least 30 m without an assistive device, (3) no history of lower-limb surgery or joint replacement, (4) no acute musculoskeletal injuries in the back or lower extremities within 6 months preceding the testing sessions, and (5) no known neurological diseases (e.g., Parkinson’s disease, stroke, or peripheral neuropathy) that would affect postural control.

Exclusion criteria included impaired cognition that precluded understanding or following task instructions, uncontrolled systemic illness, or significant visual or vestibular deficits that would affect balance performance.

The sample size was determined using G*Power 3.1.9.7 (Heinrich Heine University Düsseldorf, Düsseldorf, Germany) for a two-group comparison (α = 0.05, power = 0.80). Based on effect sizes observed in our previous studies involving IMU variables (expected range 0.25–0.40), the required total sample size was estimated to be 52–168 participants (26–64 per group). Allowing an anticipated drop-out rate of 10%, we recruited 50 participants per group, yielding a target total sample size of 100 participants.

Demographic characteristics are presented in Table 1. The knee OA group was composed of 33 women (75%) and 11 men with a mean age of 71.23 ± 5.75 years while the control group was constituted by 27 women (60%) and 18 men with a mean age of 70.87 ± 4.30 years. There was no significant difference between the two groups regarding their body weight, height or BMI. Among the patients with OA, the median Kellgren–Lawrence score was 3 (interquartile range 2 to 3), reflecting moderate and severe radiologic findings.

Patients with knee OA in our study were recruited from the PMR outpatient clinic, while controls were community-dwelling healthy older individuals selected using age-matching criteria through community centers or advertisements. All controls had no history of knee symptoms or signs. This approach provided comparable clinical settings; however, we acknowledge that recruiting OA patients from a hospital-based population may introduce selection bias, limiting the generalizability of the findings to the broader community-dwelling elderly population.

### 2.3. Inertial Measurement Unit System

**Figure 1:**
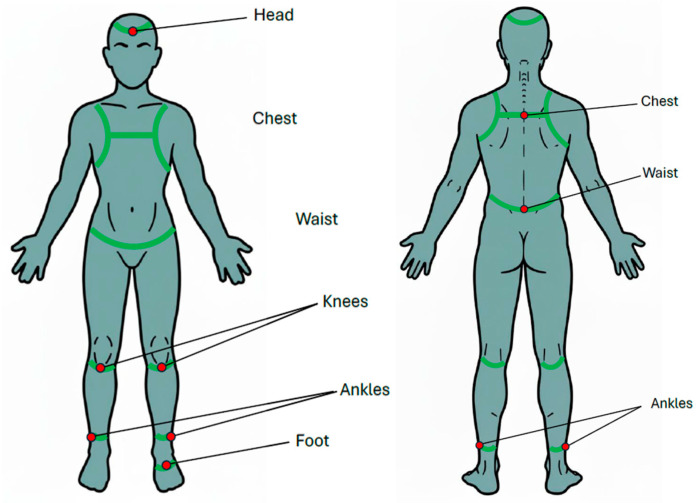
Schematic diagram showing the placement of inertial measurement units (IMUs). Green lines indicate the straps to secure the IMU sensor, and the red dots mark the sensor locations.

**Figure 2:**
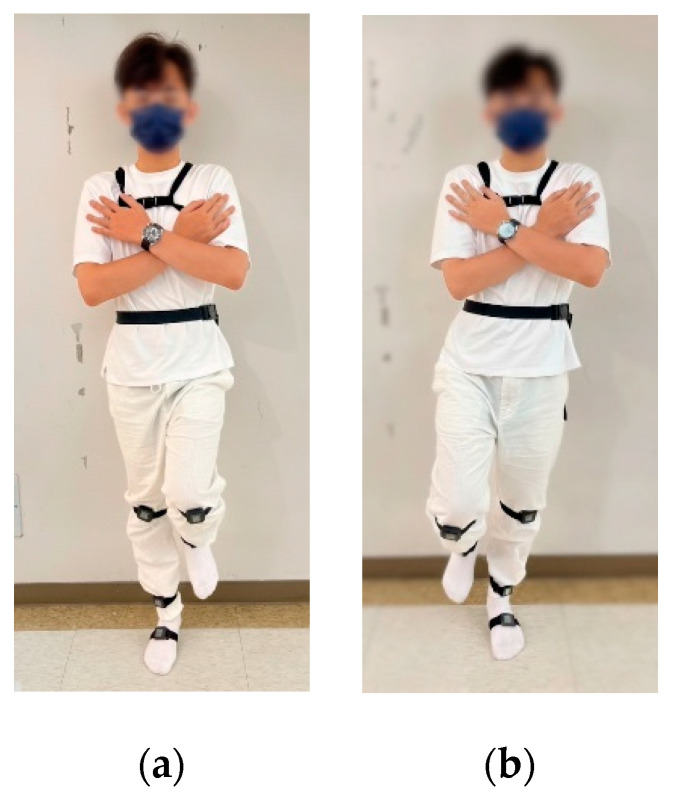
Single-leg stance test. Representative images of (**a**) right leg standing and (**b**) left leg standing.

**Figure 3:**
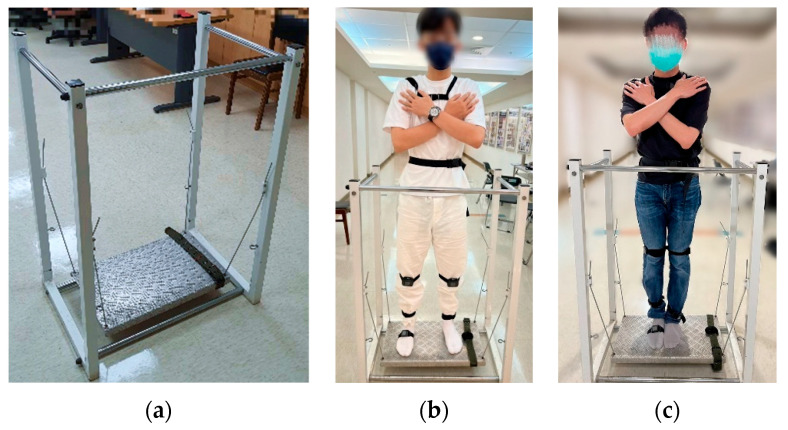
Balance board stance tests. Representative images of (**a**) the balance board; (**b**) the feet with shoulder-width apart stance and (**c**) the feet-together stance.

Kinematic data was collected using the commercial IMU system (APDM Wearable Technologies, Portland, OR, USA) [27], which comprises docking stations, access points, and wearable wireless sensors designed for motion analysis. The technical specifications of the Opal IMU sensors used in this study are summarized in Appendix A. For the normal walking test, two IMUs were affixed to chest (at the level of sternum) and waist (around the iliac crest, at the level of L5 vertebra). Diagram illustrating sensor placement was shown in Figure 1. For the single-leg stance (SLS) test, eight IMUs were affixed to head (around the forehead), chest (at the level of sternum), waist (around the iliac crest, at the level of L5 vertebra), both knees (at the distal aspect of patella), both ankles (at proximal aspect of lateral malleolus), and the sole of the standing foot (Figure 2). For the balance board stance test, an additional IMU was affixed to the balance board using a belt to record board movement for the balance board tasks (Figure 3b,c).

Three-dimensional angular velocities, linear accelerations, and Quaternion data were recorded using a commercial IMU system equipped with built-in filters to minimize the influence of artifacts at a sampling rate ranging from 20 to 128 Hz. In our study, we applied the highest sampling rate (128 Hz), which was sufficient to capture gait and balance data, as demonstrated in previous studies [28].

### 2.4. Normal Walking Test

To obtain a baseline of dynamic gait stability, all examiners were instructed to walk back and forth twice along a 6 m walkway, covering a total distance of approximately 24 m at their normal walking speed. To minimize the influence of transitional events, we analyzed only steady-state strides from the middle section of the walkway and excluded initiation and turning from the analyses. Analyses were referenced to the right foot, 16–20 of steps taken on average were used to evaluate walking performance.

Swing phase ratio (SP ratio) was calculated as the percentage of time-spend in swing phase within the gait cycle. For the analysis of interlimb coordination, the swing phase asymmetry ($Asym_{SP}$) [29] was calculated by subtracting the SP ratio of the non-affected limb (*SP_NA_*) from that of the affected limb (side with osteoarthritic changes, *SP_OA_*), dividing by affected limb SP ratio.

Swing phase asymmetry ($Asym_{SP}$) was calculated using the following formula where *SP_OA_* and *SP_NA_* denote the swing phases of the OA limb and non-affected limbs, respectively. Higher $Asym_{SP}$ values indicate greater asymmetry, reflecting reduced gait symmetry and increased risk of balance impairments.

$$Asym_{SP}=\frac{SP_{OA}-SP_{NA}}{SP_{OA}}\times 100\%$$

### 2.5. Static Balance: Single-Leg Stance Test

Static postural control was evaluated using a Single-Leg Stance (SLS) test. Participants stood barefoot on a flat, firm surface for a maximum of 60 s (Figure 2). In the knee OA group, the more symptomatic limb (based on clinical severity and radiographic findings) was selected; in controls, the corresponding limb was used. The test sequence involved standing on the right leg with the left leg elevated, followed by standing on the left leg with the right leg elevated. Participants crossed their arms over the chest to minimize upper-limb compensation and were allowed to rest between trials as needed. Participants were instructed to fixate on a target approximately one meter ahead to minimize variability from uncontrolled eye movements. Trials were terminated if balance was lost (e.g., foot contact with the ground or excessive movement) and could be repeated up to two additional times. Only the longest successful trial (up to 60 s) was included in the analysis.

The longest successful trial was selected for analysis, as it is considered to most accurately represent each participant’s maximal balance capacity rather than variability related to unfamiliarity or initial hesitation. This approach is more likely to capture intrinsic determinants of balance performance such as postural control, proprioception, and lower-limb strength, rather than procedural or motivational nuances. A 30–60 s rest period was given for the participants between trials to avoid fatigue and ensure consistent performance. If a participant was unable to maintain balance for 60 s on the initial attempt, up to two further attempts were allowed, for a maximum of three attempts in total.

### 2.6. Dynamic Balance: Balance Board Stance Test

Dynamic postural stability was assessed using a suspended balance board system (Monitored Rehab Systems, Haarlem, The Netherlands) commonly employed in clinical rehabilitation for patients with neurological conditions such as stroke or spinal cord injury.

The balance board (Figure 3a) measured 60 × 35 cm and was suspended horizontally by four high-stiffness steel cables attached to a surrounding metal frame. Handrails were placed at the front and sides for safety. The suspension system allowed horizontal movement of the board in response to participants’ body motions, generating self-induced perturbations. Because the cables were highly stiff and the board’s motion involved only small-angle sway, the suspended board was approximated as a pendulum exhibiting simple harmonic motion. This pendulum model provides a standard framework for describing how a suspended platform responds to forces and moments applied by a person standing on it. Within this framework, participants’ corrective body movements act as control inputs to stabilize the board, enabling quantification of dynamic balance ability beyond simple descriptive measures.

Participants were asked to stand on the board barefoot and to cross their arms over their chest to avoid upper limbs compensation. Two stance conditions were tested sequentially: feet shoulder-width apart (maximum 60 s) and feet together (maximum 60 s) (Figure 3b,c). Rest periods were permitted between trials, and participants could terminate the test at any time by grasping the handrails. Trials were stopped if balance was lost (e.g., excessive movement, stepping off, or using the handrails) and could be repeated up to two additional times. For analysis, only the longest successful attempt (up to 60 s) was retained.

### 2.7. Evaluation of Static Balance and Dynamic Balance

To quantitatively assess gait stability and balance performance, we defined two biomechanical indexes based on the IMU data: the average absolute angular velocity ($J_{\omega }$) and the average absolute linear acceleration ($J_{\alpha }$). These two indices were used because these IMU-based metrics demonstrated high reliability and clear age-related differences in healthy adults in our previous methodological study [20]. The indices reflect the extent of postural adjustments made by different body segments during balance tasks with formular listing below, respectively, with ω stands for angular velocity and α stands for linear acceleration:

The average absolute angular velocity for segment k was calculated as:

(1) $$J_{\omega }^{k}=\frac{1}{N}\sum_{i=1}^{N}\left|\omega^{k}(i)\right|=\frac{1}{N}\sum_{i=1}^{N}{\left((\omega_{x}^{k}(i){)}^{2}+(\omega_{y}^{k}(i){)}^{2}+(\omega_{z}^{k}(i){)}^{2}\right)}^{1/2}$$

$where\;k\in \left\{head,chest,\;waist,knee,\;ankle,\;sole\;of\;the\;foot,balance\;board\right\}$ indicates the body segment or device. $\omega_{x}^{k}\left(i\right),\;\omega_{y}^{k}\left(i\right),\;\mathrm{and}\omega_{z}^{k}\left(i\right)$ are the tri-axial angular velocity components recorded by the IMU at location k during the *i*-th sampling point, and N is the total number of samples in the trial. The subsequently calculated $J_{\omega }^{k}$, quantifies the average degree of rotational activity, with higher values reflecting greater compensatory angular adjustments to maintain postural stability. Angular velocity is reported in radians per second (rad/s).

The average absolute linear acceleration for segment k was calculated as:

(2) $$J_{\alpha }^{k}=\frac{1}{N}\sum_{i=1}^{N}\left|\alpha^{k}(i)\right|=\frac{1}{N}\sum_{i=1}^{N}{\left((\alpha_{x}^{k}(i){)}^{2}+(\alpha_{y}^{k}(i){)}^{2}+(\alpha_{z}^{k}(i){)}^{2}\right)}^{1/2},$$

where $\alpha_{x}^{k}\left(i\right),\;\alpha_{y}^{k}\left(i\right),\;\mathrm{and}\alpha_{z}^{k}(i)$ are the linear acceleration components recorded by the IMU at location k during the *i*-th sampling point. $J_{\alpha }^{k}$ quantifies the average degree of translational activity, with higher values reflecting greater compensatory linear adjustments to maintain postural stability. Linear acceleration is reported in meters per second squared (m/s^2^).

Although both $J_{\omega }^{k}\;$ and $J_{\alpha }^{k}$ characterize balance strategy, prior work [20] and our pilot data suggest that $\;J_{\omega }^{k}$ may be a more sensitive measure of compensatory postural adjustments. Both indices were computed under static (single-leg stance) and dynamic (balance board) conditions to yield a comprehensive balance performance profile. By comparing knee OA and control groups, we sought to identify compensatory patterns associated with joint degeneration and postural instability.

### 2.8. Statistical Analysis

All analyses were performed using SPSS Statistics version 22.0 (IBM Corp., Armonk, NY, USA). We performed a multivariate analysis of variance (MANOVA) with group as the factor and body-part indices as dependent variables. This approach accounts for the relationship between outcomes and avoids increasing Type I errors. For univariate tests requiring adjustment, Bonferroni-adjusted *p*-values were applied to conduct a conservative sensitivity analysis. Results are reported as mean ± standard deviation (SD). Statistical significance was set at *p* < 0.05.

## 3. Results

### 3.1. Demographic Data

A total of 89 participants were recruited and allocated into two groups: adults 44 or older with clinically diagnosed knee OA (mean age ± SD [range]): 71.23 ± 5.75 years [61–86]) and 45 age-matched healthy controls (70.87 ± 4.30 years [62–84]). Table 1 summarizes the demographic characteristics of both groups. No significant differences were observed between the groups regarding age (*p* = 0.742), body weight (*p* = 0.401), height (*p* = 0.569), or body mass index (*p* = 0.633), indicating successful matching of key physical variables.

The proportion of women was higher in the knee OA group (75%) than in the control group (60%), which is consistent with the higher prevalence of knee OA among females [30]. Regarding functional performance, the mean TUG test duration was 8.79 ± 1.74 s in the knee OA group and 9.22 ± 2.13 s in the control group (*p* = 0.337). Within the knee OA group, the median Kellgren–Lawrence grade was 3 (interquartile range (IQR): 2–3), reflecting moderate-to-severe radiographic changes [31]. Collectively, these results confirm that the knee OA and control groups were comparable in demographic and functional characteristics, apart from the expected sex distribution.

### 3.2. Evaluation of Gait Performance

Gait performance was evaluated using multiple kinematic parameters derived from the IMU sensors during the normal walking test. These included swing phase asymmetry ($Asym_{SP}$) [29], swing phase ratio (SP ratio) and segmental measures of average absolute angular velocity ($J_{\omega }$), as well as average absolute linear acceleration ($J_{\alpha }$) at the chest and waist segments.

**Figure 4:**
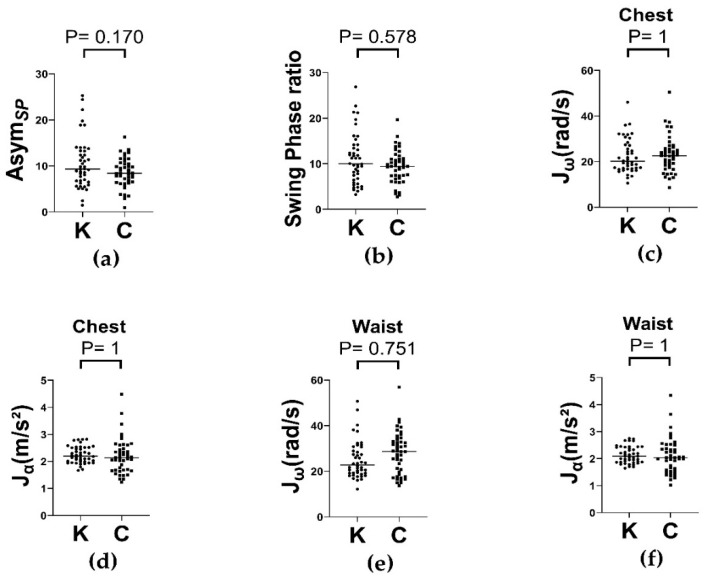
Gait performance during normal walking test in knee OA (K) and control (C) groups, with medians indicated. (**a**) Swing phase asymmetry in Knee OA and Control groups. (**b**) Swing phase ratio in Knee OA and Control groups. (**c**,**d**) Average absolute angular velocity ($J_{\omega }$) and Average absolute linear acceleration ($J_{\alpha }$) recorded at the chest, respectively. (**e**,**f**) $J_{\omega }$ and $J_{\alpha }$ recorded at the waist, respectively. $J_{\omega }$: Average absolute angular velocity; $J_{\alpha }$: Average absolute linear acceleration.

The $Asym_{SP}$ index was higher in the knee OA group compared with controls (10.57 ± 5.48 versus 8.45 ± 3.21, *p* = 0.170; Figure 4a), but this difference was not significant, and the SP ratio also showed no group difference (Figure 4b). Likewise, $J_{\omega }$ and $J_{\alpha }$ at the chest and waist likewise did not differ between groups (Figure 4c–f), although a trend toward greater trunk rotational movement (higher $J_{\omega }$ values) was observed in the knee OA group.

These findings suggest that older adults with knee OA may not display marked gait asymmetry or trunk kinematic changes during low-demand, level walking, particularly in the absence of advanced disease [32,33].

### 3.3. Evaluation of Postural Stability: Single-Leg Stance Test

**Figure 5:**
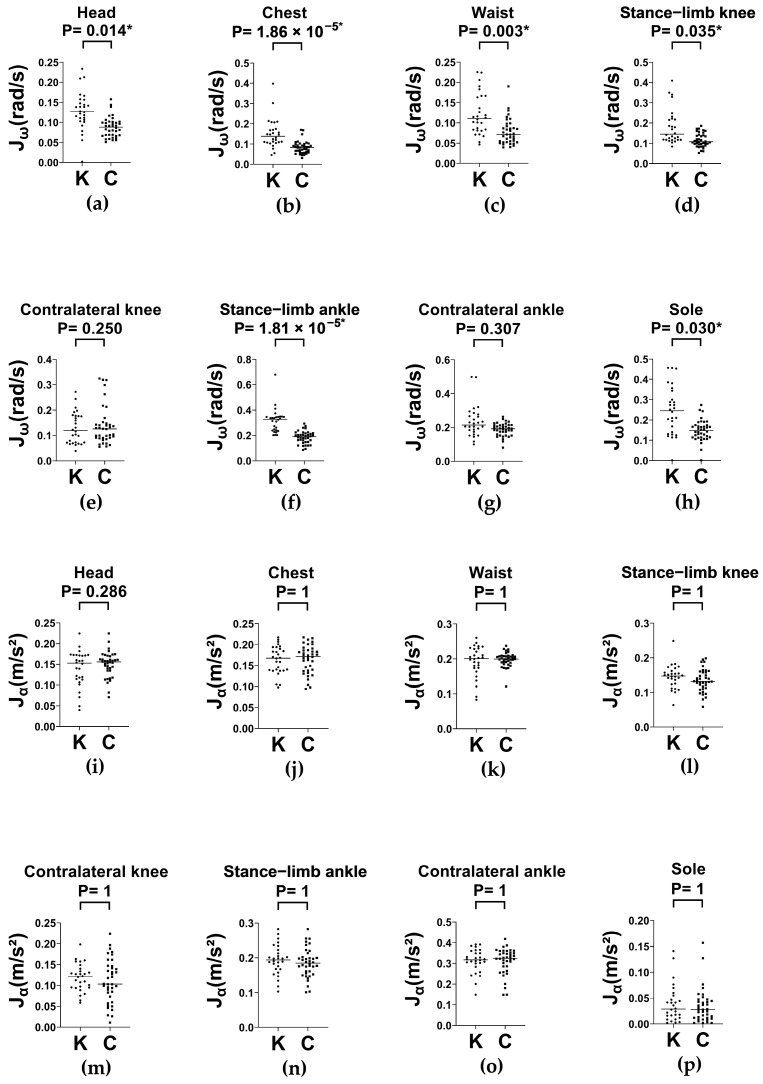
Column scatter plots (with medians) of $J_{\omega }$ (**a**–**h**) and ${\;J}_{\alpha }$ (**i**–**p**) across body segments during single-leg stance test in knee OA (K) and control (C) groups. $J_{\omega }$: average absolute angular velocity; $J_{\alpha }$: average absolute linear acceleration. * *p* < 0.05.

We placed IMUs on multiple body segments to quantify static balance performance during the SLS test. Analyses of the average absolute angular velocity $J_{\omega }$ are presented in Figure 5.

Compared with controls, the knee OA group exhibited greater $J_{\omega }$ across most measured segments (Figure 5a–d,f,h). Group differences were statistically significant at the head (*p* = 0.014), chest (*p* = 1.86 × 10^−5^), waist (*p* = 0.003), and stances, including limb knee (*p* = 0.035), limb ankle (*p* = 1.81 × 10^−5^), and limb sole (*p* = 0.030). A per-segment summary for group means ± SD of both $J_{\omega }$ and $J_{\alpha }$ during SLS is presented in Appendix B. Increased rotational motion in the knee OA group was more pronounced in the proximal segments (chest and waist) and limb joints, suggesting compensatory strategies for maintaining postural stability. No significant group difference was observed in the contralateral knee and ankle, indicating that postural adjustments during the static stance primarily involve the trunk and stance limb rather than the contralateral lower limb.

These findings support the hypothesis that older adults with knee OA have impaired static postural control, as reflected by increased angular movement in critical postural segments. Moreover, the results highlight $J_{\omega }$ as a sensitive and discriminative marker for balance instability.

Average absolute linear acceleration ($J_{\alpha }$) across body segments was also examined. As shown in Figure 5i–p, none of the segments showed statistically significant group differences included. Collectively, these findings suggest that $J_{\alpha }$ is less sensitive than $J_{\omega }$ in detecting postural control deficits during single-leg stance. Overall, the broadly similar translational acceleration patterns between groups indicated limited displacement of the body’s center mass, whereas angular metrics revealed more pronounced postural compensation.

### 3.4. Evaluation of Dynamic Balance: Balance Board Stance Test

**Figure 6:**
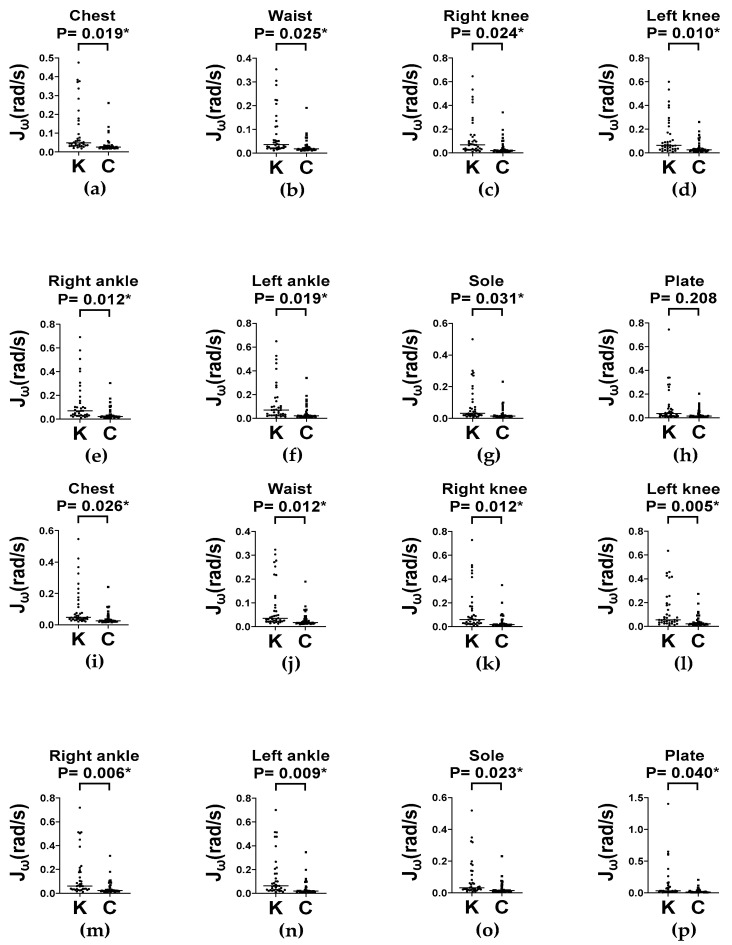
Column scatter plots (with medians) of $J_{\omega }$ across body segments during the first 30 s (**a**–**h**) and 60 s (**i**–**p**) of balance board stance with feet-apart in knee OA (K) and control (C) groups. $J_{\omega }$: average absolute angular velocity. * *p* < 0.05.

**Figure 7:**
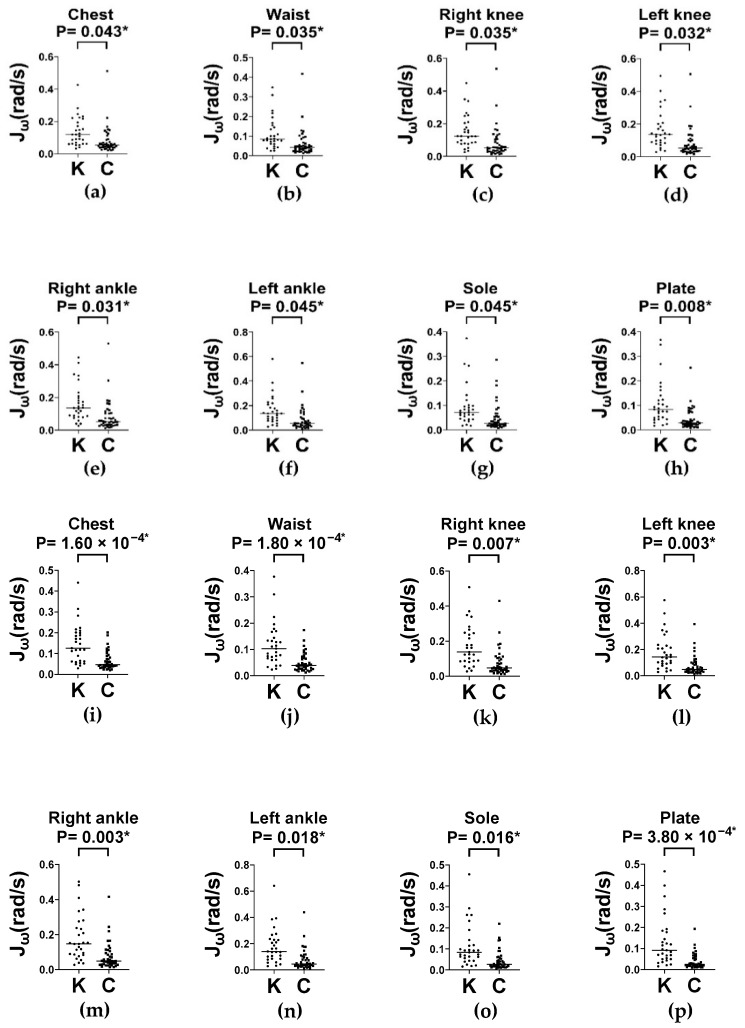
Column scatter plots (with medians) of $J_{\omega }$ across body segments during the first 30 s (**a**–**h**) and 60 s (**i**–**p**) of balance board stance with feet-together in knee OA (K) and control (C) groups. $J_{\omega }$: average absolute angular velocity. * *p* < 0.05.

Dynamic postural control was evaluated using the balance board stance test under two stance conditions (feet apart and feet together) at two time intervals (0–30 s for initial responses and 0–60 s for sustained performance). In the feet-apart stance, the knee OA group exhibited significantly greater segmental angular velocity ($J_{\omega }$) across all measured body segments compared to controls, at both 0–30 s and 0–60 s (Figure 6). These differences are evident in the chest, waist, bilateral knees and ankles, and sole (all *p* < 0.05). A similar pattern was observed in the feet-together stance, where the knee OA group consistently exhibited higher $J_{\omega }$ values across both intervals (Figure 7).

These findings suggest that individuals with knee OA make more frequent or larger postural adjustments, potentially reflecting compensatory response to impaired neuromuscular control or proprioceptive deficits. The persistent elevation of ${\;J}_{\omega }$ across both time intervals indicates deficits in both anticipatory and sustained balance control, which appears to be further challenged in the feet-together stance due to the reduced base of support.

**Figure 8:**
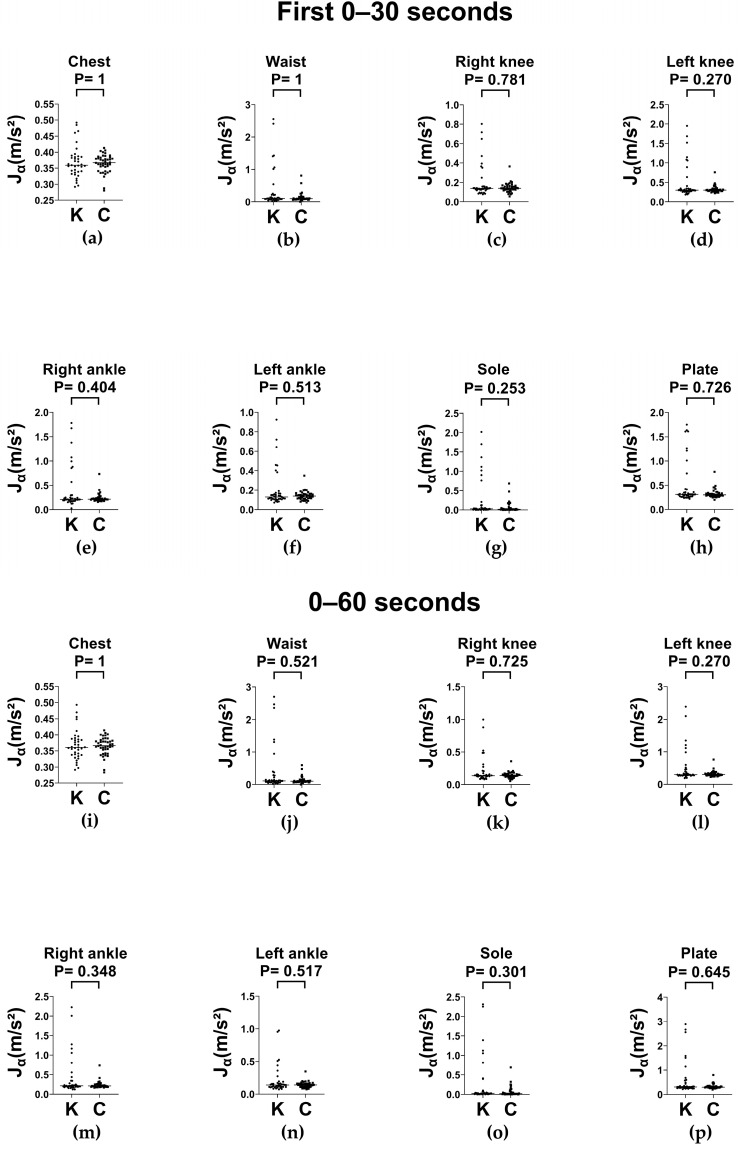
Column scatter plots of $J_{\alpha }$ (with medians) of the balance board stance across body segments with feet-apart, in knee OA (K) and control (C) groups. (**a**–**h**) Average absolute linear acceleration ($J_{\alpha })$ recorded at the chest, waist, right knee, left knee, right ankle, left ankle, sole, and plate respectively during 0–30 s interval. (**i**–**p**) Average absolute linear acceleration ($J_{\alpha })$ recorded at the chest, waist, right knee, left knee, right ankle, left ankle, sole, and plate respectively during 0–60 s interval. $J_{\alpha }$: average absolute linear acceleration.

**Figure 9:**
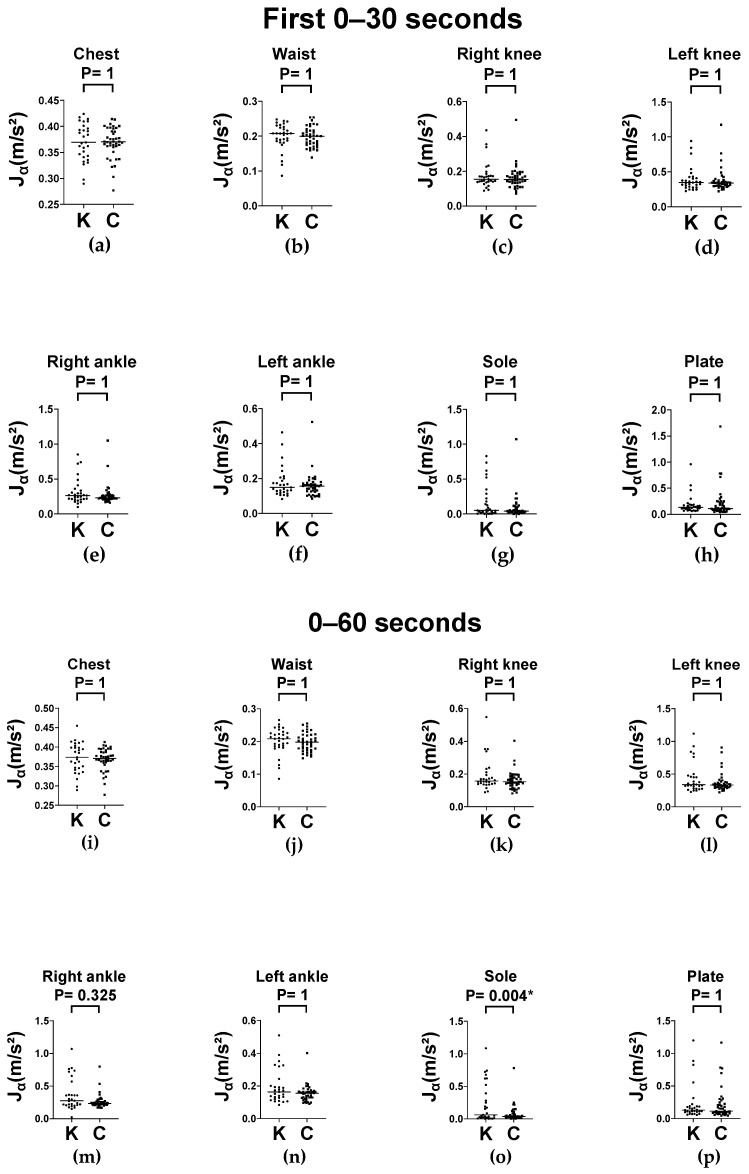
Column scatter plots of $J_{\alpha }$ (with medians) of the balance board stance with feet-together in knee OA (K) and control (C) groups. (**a**–**h**) Average absolute linear acceleration ($J_{\alpha })$ recorded at the chest, waist, right knee, left knee, right ankle, left ankle, sole, and plate respectively during 0–30 s interval. (**i**–**p**) Average absolute linear acceleration ($J_{\alpha })$ recorded at the chest, waist, right knee, left knee, right ankle, left ankle, sole, and plate respectively during 0–60 s interval. $J_{\alpha }$: average absolute linear acceleration. * *p* < 0.05.

Regarding average absolute linear acceleration ($J_{\alpha }$), no significant differences were observed between groups across stance conditions or time windows (Figure 8 and Figure 9, respectively) in almost all parts, except for the sole segment of balance board stance with feet-together at 0–60 s interval. The higher $J_{\alpha }$ from the sole in knee OA is probably a distal compensation strategy and reflects an increased ankle–foot adaptation in compensation to maintain balance despite impaired proximal control. In addition, measurement artifacts (sensor displacement in soft tissues or interaction of foot and plate) may result in overestimated acceleration values even if the abovementioned joint kinematics are similar. These findings indicate that $J_{\alpha }$ may be less sensitive than $J_{\omega }$ for detecting segmental instability during dynamic balance tasks.

Thus, $J_{\omega }$ may be a more sensitive and discriminative measure for dynamic balance impairment for the at-risk group for a given segment involving both the lower extremity and trunk compared to $J_{\alpha }$. This further strengthens its utility as an evaluative outcome measure that can be informative in clinical assessments and intervention planning for balance dysfunction in older populations.

## 4. Discussion

In our study, we evaluated the dynamic and static postural control of older adults with knee OA using a wearable IMU system during level-ground walking and various balance tasks. Participants with knee OA showed significantly greater average absolute angular velocity ($J_{\omega }$) across nearly all body segments during both the single-leg stance and balance board conditions. These effects were consistent across different foot placements (feet apart and feet together) at two time intervals (0–30 and 0–60 s), indicating the presence of postural instability in the initial phase that was sustained over time. In contrast, average absolute linear acceleration ($J_{\alpha }$) did not significantly differ between groups for the conditions considered. Additionally, gait symmetry and trunk kinematics did not differ between the knee OA and control groups during level ground walking. These findings suggest that knee OA is associated with excessive multi-segmental compensatory movements in response to postural perturbations, validating angular velocity as a sensitive metric for detecting postural instability in this population.

Various dynamic platforms have been used to assess dynamic balance [34,35], including wobble boards [36,37] and computerized dynamic posturography (CDP) [38,39]; however, wobble boards often lack safety handrails, posing risks for older adults while CDP systems are costly and mostly confined to laboratory settings. The suspended balance board in our study provided both safety and experimental control, making it suitable for older adults, as shown in our previous study [20], and for individuals with knee OA.

As kinematic metrics, the angular velocity and linear acceleration provide a segmented perspective on how individuals adapt to postural demands. Specifically, angular velocity quantifies the rotatory movements of body segments around their axes, directly reflecting neuromuscular compensation such as trunk rotation, limb swing, or body sway induced by balance perturbation. In line with the findings of Alsubaie et al. [40], who demonstrated that the root mean square of trunk angular velocity outperforms angular displacement and linear acceleration in the assessment of postural control, our results support the robustness and reliability of angular velocity measurements in clinical populations. Individuals with knee OA often use compensatory strategies that involve multiple body segments to maintain stability in the presence of joint instability or proprioceptive deficits [41]. Therefore, segmented angular velocity metrics provide critical insights into these adaptive mechanisms. In contrast, linear acceleration primarily captures unidirectional random movements, which despite being informative, may be less sensitive to the subtle multidirectional postural adjustments required for maintaining balance during regulated standing tasks. Although the OA group exhibited higher linear acceleration values in regions such as the waist and knees, the moderate variability in this parameter limits its clinical utility in clearly distinguishing balance dysfunctions across individuals. Collectively, these findings advance the objective assessment of postural control deficits in knee OA and highlight angular velocity as a priority measure for identifying postural instability. Our previous investigations successfully differentiated balance performance among different age groups using similar IMU-based angular velocity analyses [20]. Overall, the integration of multiple wearable IMUs combined with the refined signal processing of angular velocity data offers a comprehensive and nuanced understanding of the complex balance adaptations exhibited by clinical populations.

Exploring balance adaptation over time by examining both the initial 0–30 s and the full 0–60 s of each balance task provided valuable insights into the postural adaptation mechanism. Le Clair and Riach [42] showed that 20 and 30 s trials yield the highest test–retest reliability in quiet standing, noting that a 10 s trial is insufficient for capturing representative sway. Similarly, Doyle et al. [43] found that in the eyes-open condition, the center of pressure velocity achieved adequate reliability in the two 30 s trials, whereas spatial parameters required at least five 60 s trials. Accordingly, we selected the 0–30 s period to capture anticipatory preparation and immediate response postural instability, where the 0–60 s interval reflected the participants’ ability to sustain postural strategies and counteract destabilizing influences potentially caused by fatigue. Our results demonstrated significant differences in angular velocity during the initial 30 s of both balance board tasks, with the differences persisting or increasing over the full 60 s duration. This temporal pattern suggests that individuals with knee OA experience impairment in both anticipatory (early phase) and sustained (long-lasting) postural control. These findings underscore the clinical importance of incorporating multiphase observation periods when assessing balance.

Conventional balance assessment tools such as the TUG test [7], Berg balance scale [8], and functional reach test [44] primarily yield categorical or coarse-grain data. Although these tools are clinically valuable, they often fail to capture the subtle compensatory behaviors and segmental movement patterns underlying balance impairments. By contrast, our IMU-based analysis provides a continuous, quantitative measurement of segmental kinematics, allowing the detection of subtle joint realignments that may reflect neuromuscular control strategies that are not readily visible in typical clinical evaluations. This suggests that kinematic indices derived from wearable sensors have the potential to complement traditional assessment methods and enhance the accuracy of balance evaluation. Supporting this notion, previous research has demonstrated that wearable IMUs exhibit moderate-to-good intra-trial reliability (ICC = 0.50–0.67) and good inter-trail reliability (ICC = 0.75–0.86) in measuring postural sway [45], further validating their utility in both clinical and research settings.

Previous research using posturography showed that patients with knee OA exhibit a larger medial-lateral center of pressure range during quiet standing [46,47]. Another study using computerized dynamic posturography demonstrated that elderly women with unilateral knee OA have impaired postural balance, as evidenced by greater sway during one-leg standing, reduced control during weight shifts, a slower rise from sit to stand, and longer step-up times [12]. However, computerized dynamic posturography is equipment-intensive and lab-based and may miss detailed segment-level motion analysis. Segment-level compensatory movements in knee OA involve the biomechanical adaptation of various joints and muscle groups, including the trunk and ankle, which help reduce joint loading and pain by modifying joint angles and muscle activation patterns. These compensations help decrease medial compartment loading but can cause other biomechanical effects, making it challenging to measure without detailed motion analysis. Recognizing these factors is critical for designing effective rehabilitation strategies tailored to individual biomechanical adaptations of patients with knee OA. Furthermore, clinical scales such as Community Balance and Mobility, which assess functional balance and mobility can distinguish between patients and controls despite lacking sensitivity to subtle segment-specific adaptations because of their ordinal nature [10].

Our study builds on these tools and complements them by placing IMUs at different body locations to quantify the trunk and distal kinematics during balance tasks. We derived two indices: average absolute angular velocity ($J_{\omega }$) and average absolute linear acceleration ($J_{\alpha }$). Previous research on knee OA balance has mainly focused on center-of-pressure metrics or clinical scales rather than on segment-resolved wearable sensors. Our study is among the first to integrate multi-segment IMUs with single-leg stance and balance board paradigms in older adults with knee OA, providing a scalable, clinically useful approach that pinpoints where (trunk versus distal joints) and when (early versus sustained) compensatory strategies occur.

However, this study has some limitations. First, although our sample size was sufficient to identify statistically significant differences in key outcome measures, the cohort comprised only older adults without major cognitive or neurological comorbidities. Therefore, the transferability of these findings to broader clinical populations, including those at higher risk of falling or with multi-joint involvement, remains limited. Second, selecting the more symptomatic leg in the knee OA group may introduce variability due to subjective interpretation or disparities in radiographic severity between the limbs [48]. Third, although IMUs provide valuable spatiotemporal kinematic data, they do not directly measure neuromuscular activation, joint torque, or sensorimotor integration. Electromyography can complement IMUs by capturing the underlying neuromuscular function, which is essential for a comprehensive understanding of gait mechanics. Consequently, the compensatory strategies inferred from angular velocity alone may not fully represent the underlying biomechanical or neural mechanisms. Fourth, the absence of systematic monitoring or control of arm motion may have introduced unmeasured variability in upper limbs contributions to mediolateral and anteroposterior balance, especially during transitions (e.g., initiation of gait or turning). Fifth, we did not implement an imaging-based compartment classification or frontal alignment to allow stratification of analyses of the effects per-compartment which may affect gait mechanics and balance outcomes. Sixth, the minimum stance time cutoff of 10 s for the inclusion analysis may have excluded participants with greater balance deficits, potentially underestimating postural instability in the more impaired subgroups. Finally, normative reference data for the indices $J_{\omega }$ and $J_{\alpha }$ are lacking in routine clinical practice; therefore, the interpretation relied on relative comparisons rather than absolute thresholds. Hence, future studies are required to establish validated clinical benchmarks by integrating these metrics into fall risk evaluations and rehabilitative practices.

Future research should explore additional physiological inputs, such as visual occlusion and joint proprioception tests, to delineate the sensory contributions to balance control [49]. We also aim to examine the sensitivity of $J_{\omega }$ to interventions, such as trunk stabilization and lower-limb proprioception training. These perturbation-based studies can account for individual compensation strategies in knee OA, setting the foundation for advanced sensor-based rehabilitation protocols that enable continuous segmental movement monitoring and real-time performance feedback, to improve function and prevent falls in patients with degenerative joint disease.

## 5. Conclusions

In this study, we assessed the gait performance and static and dynamic balance in older adults with knee OA compared with age-matched controls using wearable IMUs. By analyzing average absolute angular velocity ($J_{\omega }$) and average absolute linear acceleration ($J_{\alpha }$) across multiple body segments and time intervals, we showed that the knee OA group exhibited greater angular movement during balance tasks, reflecting impaired anticipatory control and reduced postural stability. Among the extracted parameters, angular velocity was the most sensitive and reliable for detecting balance deficits. These findings support the use of IMU-based angular velocity analysis as a practical clinical tool for identifying postural instability in individuals with degenerative joint pathologies. Incorporating such objective measures into balance assessments may allow for the earlier detection of instability and guide more targeted rehabilitation strategies for the aging population with knee OA.

## Figures and Tables

**Table 1:** Demographic data.

Characteristics	Osteoarthritis Group	Control Group	*p*-Value
Number, n	44	45	-
Age, years	71.23 ± 5.75	70.87 ± 4.30	0.742
Female, n (%)	33 (75%)	27 (60%)	-
Weight, kg	61.23 ± 10.02	63.25 ± 12.40	0.401
Height, cm	158.82 ± 9.19	159.93 ± 9.23	0.569
Body mass index	24.25 ± 3.24	24.60 ± 3.67	0.633
TUG Test, seconds	8.79 ± 1.74	9.22 ± 2.13	0.337
Osteoarthritis Kellgren-Lawrence grade	3 (2–3) *	-	-

Data are number (%) or mean ± SD. * Median (IQR).

## Data Availability

The clinical data sets during this study are not publicly available because of ethics and privacy. De-identified data can be made available upon request to the corresponding author with the approval of the Institutional Review Board and a data use agreement.

## Appendix A

The specifications of the IMU system are available at: https://reurl.cc/davdo8 (accessed on 6 October 2025).

## Appendix B

The per-segment description for group means ± SD of both $J_{\omega }$ and $J_{\alpha }$ during SLS tests are available at: https://reurl.cc/z5nNme (accessed on 6 October 2025).
